# Using general practitioners to recruit individuals with low socioeconomic position to preventive health checks is feasible: a cross sectional study

**DOI:** 10.1080/02813432.2019.1639901

**Published:** 2019-07-09

**Authors:** Nina Kamstrup-Larsen, Susanne Oksbjerg Dalton, Marie Broholm-Jørgensen, Lars Bruun Larsen, Janus Laust Thomsen, Christoffer Johansen, Janne Schurmann Tolstrup

**Affiliations:** a National Institute of Public Health, University of Southern Denmark, Copenhagen, Denmark;; b Survivorship Research Unit, the Danish Cancer Society Research Center, Copenhagen, Denmark;; c Department of Clinical Oncology and Palliative Care, Zealand University Hospital, Naestved, Denmark;; d Research Unit of General Practice in Odense, University of Southern Denmark, Denmark;; e Research Unit for General Practice in Aalborg, Department of Clinical Medicine, Aalborg University Hospital, Aalborg, Denmark;; f Late Effect Research Unit CASTLE, Finsen Center, Rigshospitalet, Copenhagen, Denmark

**Keywords:** Health check, uptake, non-attendance, general practitioner, social inequality, socioeconomic factors, prevention

## Abstract

**Objective:** To test whether demographic and health-related characteristics are associated with non-attendance of preventive health checks offered to individuals with low levels of education using proactive recruitment by the general practitioners.

**Design:** A cross-sectional study.

**Setting:** 32 general practice clinics in Copenhagen, Denmark.

**Subjects:** A total of 549 individuals aged 45–64, with low levels of education, enrolled in the intervention group of a randomised controlled trial on preventive health checks offered by general practitioner.

**Main outcome measures:** Non-attendance of the preventive health checks.

**Methods:** (i) Descriptive characteristics of attendees and non-attendees and (ii) crude and adjusted multi-level logistic regression to examine associations of individual characteristics with non-attendance of preventive health checks.

**Results:** Overall, 33% did not attend the prescheduled preventive health checks at their general practitioners. Non-attendees were more likely to live without a partner, be of non-Western origin, be daily smokers, have poor self-rated health, have higher pulmonary symptoms score, have increased level of stress, have low levels of self-efficacy, have metabolic risk factors or non-communicable diseases and have had no contact with their general practitioner within the past year.

**Conclusion:** The findings suggest that, it is feasible to use general practitioners for recruiting individuals for preventive health checks. However, even in a trial targeting individuals with low levels of education, there are differences between attendees and non-attendees, with a more adverse health behaviour profile and worse health status observed among the non-attendees.

**KEY POINTS**
Current awareness
• Non-attendance of preventive health checks offered to the general population is associated with low socioeconomic position and adverse health behaviours.
Main statements
• It is feasible to use general practitioners proactively in recruitment to preventive health checks offered to individuals with low socioeconomic positions.
• In a trial targeting individuals with low levels of education, there were differences between attendees and non-attendees.
• Non-attendance was associated with daily smoking, poor self-rated health, high stress and no contact with the general practitioner within the last year.

## Introduction

Non-attendance among individuals with low socioeconomic positions (SEP) is a known challenge in connection with preventive health checks and is commonly mentioned as one of the reasons for the lack of population level effects seen in many studies [1–3]. Adverse health behaviours such as smoking, unhealthy diets, and risk conditions such as high blood pressure, cholesterol and blood sugar levels are associated with non-attendance of health checks [4]. These findings suggest that the inverse care law, stating that “The availability of good medical care tends to vary inversely with the need for it in the population served” [5], also apply to preventive health checks.

The Check-In randomised controlled trial (RCT) was developed to assess the effect of general practice-based health checks on health behaviour and incidence of metabolic risk factors and non-communicable diseases (NCDs). The trial targeted individuals with low SEPs, because of the lower participation rate in health checks among this group [1,2] and the higher prevalence of modifiable adverse health behaviours and NCDs in individuals with low SEPs compared to the general population [6]. One way to reach individuals with low SEPs can be to involve the general practitioners (GPs) in the recruitment. In Denmark, GPs act as gatekeepers to secondary care [7], and visits to GPs are free of charge [8]. Nearly all Danish citizens are listed with a general practice [8] and more than 80% of the population consult their GP every year [8]. General practice is characterised by continuity of care; giving the GPs a trusted position, which are found to be important in the clinical encounter [9] and highly valued by the patients [10]. Thus, general practice is a unique setting for recruiting individuals from all SEPs and was therefore used in the Check-In RCT. Furthermore, the invitations to the prescheduled preventive health check were sent out from the GPs, taking advantage of the superior evidence from proactive approaches compared to reactive approaches, e.g. in recruitment to smoking cessation programmes [11].

The objective of the present study was to test whether demographic and health-related characteristics were associated with non-attendance of general practice-based preventive health checks offered to individuals with low SEPs using proactive recruitment by the GPs.

## Material and methods

In the present study we used a cross-sectional design to analyse individual characteristics among attendees and non-attendees in a sample of 549 individuals aged 45–64 years invited to a preventive health check in the Check-In RCT. The Check-In RCT was a two-arm 1:1 trial conducted in Copenhagen, Denmark from January 2014 to September 2016 [12].

Using the unique personal identification number assigned to all residents in Denmark, we linked to individual level data in the Danish administrative registers [13–16].

### Identifying the study population

All 126 general practice clinics in four different suburbs of Copenhagen, Denmark, were invited to participate in the Check-In RCT. In total, 32 general practice clinics, including 56 GPs, agreed to participate. The patient inclusion criterion for the Check-In RCT was no formal education beyond lower secondary school, defined as no more than 11 years of schooling (Supplementary 1). As Danish GPs do not systematically record their patients’ educational level and as information from Danish registers only are available encrypted [17], educational level was obtained from questionnaires from the patients. From the patient lists of the participating GPs, 17,063 patients aged 45–64 were identified and sent questionnaires by regular mail.

The Danish questionnaire included questions about educational level, health behaviour, pulmonary symptoms, quality of life, perceived stress, self-efficacy and family dispositions of chronic diseases. Moreover, individuals were asked to indicate whether they would consent to be contacted for participation in a future research project – only individuals who reported no education beyond lower secondary school and who consented were enrolled in the trial. No exclusion criteria were applied. The questionnaire was accompanied by a short letter from the GP and the research team explaining that the questionnaire information would be entered into the electronic patient record at the GPs, and that the information could be used in future clinical encounters. Furthermore, the letter explained that participation was voluntary and would not have negative consequences for the future doctor-patient relationship. Non-responders received a reminder 3 weeks after the first questionnaire. No further action was taken to contact non-responders.

**Figure 1.**
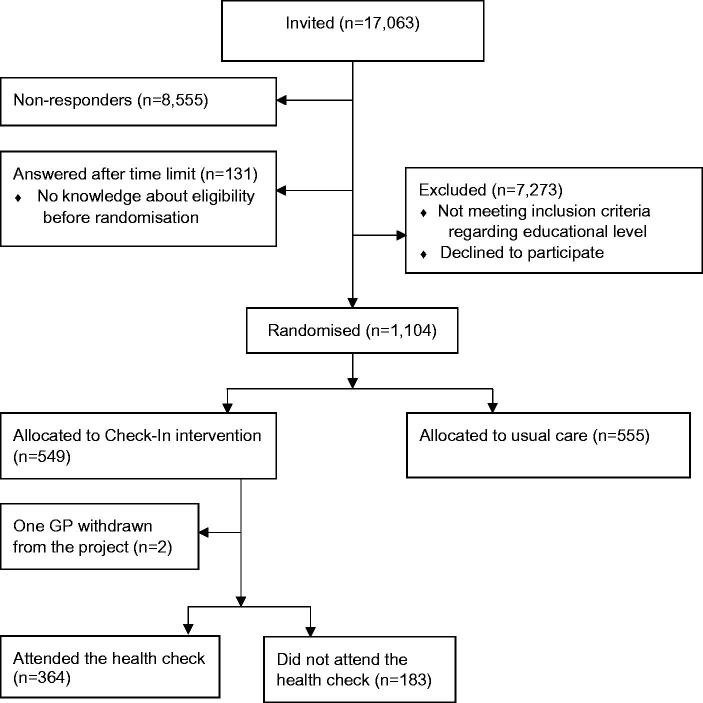
Chart showing the flow in the recruitment for the Check-In randomised controlled trial.

Overall, 49% (*n* = 8377) responded to the questionnaire. Of the responders, 1104 individuals, reported no formal education beyond lower secondary school and indicated in the questionnaire that they were willing to be contacted for further research. These individuals were enrolled in the Check-In RCT; 555 were allocated to usual care (control group) and 549 to the Check-In intervention group (Figure 1).

### The Check-In intervention

All individuals allocated to the intervention group received a personal postal invitation to a prescheduled health check from their GP, including a written description of the project by the research team. The letter clarified that participation in the study was voluntary and that individuals could withdraw at any time. Three days before the prescheduled appointment, individuals in the intervention group were reminded by phone by a member of the research team. The health check took place at the general practice clinic which the patient was listed with and was conducted by either the GP or other health staff at the clinic according to the usual clinical practice. The health checks included measurements of weight and height, hip and waist circumference, blood pressure, a blood sample for measuring serum cholesterol, HbA1c (glycated haemoglobin), thyroidal status and spirometry for smokers or former smokers. At the end of the health check a health consultation was booked. At the health consultations the GPs reviewed the results from the health checks and the questionnaires and, if necessary, arranged for further action. Individuals with abnormal results from the health check or adverse health behaviours amenable to intervention received an offer of referral to the municipal health centre for a health behaviour change programme and/or further diagnostic work-up and medical treatment. Furthermore, these individuals were offered an additional health check 6 months later.

### Measurement of variables

The main outcome was attendance and non-attendance of the preventive health check.

Information on age, affiliation to the labour market, country of origin and cohabitation status was obtained from registries administered by Statistics Denmark. Age was categorised in 5-year intervals. Affiliation to the labour market was defined as the occupational status the year before the baseline questionnaire was sent, and categorised into employed, unemployed/social benefits recipient or retired/other. Country of origin was dichotomised into Western or non-Western origin. Cohabitation status was defined as living with or without a partner. Educational level for non-responders were obtained from the Danish education registers [13]. Information on contact with the GPs was obtained from the Danish National Health Service Register [14]. Contact was defined as either a face-to-face appointment or telephone consultations in the year before the questionnaire was sent, and the variable was categorised as yes or no. Metabolic risk factors and non-communicable diseases (NCDs) were assessed as any hospital in- or outpatient contact and/or prescription medication for hypertension, hypercholesterolemia, chronic obstructive pulmonary disease (COPD), type-2-diabetes mellitus, thyroid disease and depression using the Danish National Patient Register [15] and the Danish Prescription Registry [16].

Information on health behaviour was obtained from the questionnaire conducted at baseline in the Check-In RCT. Smoking status was dichotomised into daily smoker versus not daily smoker. Alcohol consumption was reported for a normal week and divided into high-risk consumption, defined as 14/21 units of alcohol per week or more for women and men respectively [18]. BMI was generated from the self-reported height and weight and categorised into underweight (<18.5 kg/m^2^), normal weight (18.5–24.9 kg/m^2^), overweight (25–29.9 kg/m^2^), and obese (≥30 kg/m^2^) [19]. As only 24 individuals were underweight, the two lower categories were collapsed. Self-rated health was assessed by the first item in the 12-Item Short Form Health Survey (SF-12) [20] and dichotomised into good/very good/excellent and fair/poor. Pulmonary symptoms were assessed by the COPD Population Screener (COPD-PS) [21] and dichotomised with score sum ≥3 as the cut-off value (Supplementary 1). Stress during the past month was assessed by the perceived stress scale (PSS) (score range 0–40) [22]. A test for linearity showed no linearity between PSS and non-attendance and PSS was therefore dichotomised using the median as split (PSS score = 16). The person’s belief in their innate ability to achieve goals was assessed using general self-efficacy (score range 10–40) [23]. The association between self-efficacy and non-attendance was not linear and self-efficacy was dichotomised using the median as split (self-efficacy score = 29).

### Statistical analysis

Two individuals were excluded from the primary analysis as their GP withdrew from the study before the start of the intervention. Thus, 547 individuals were invited to prescheduled preventive health checks (Figure 1).

A fitted 2-level model with individuals nested within general practice clinics was conducted. Multi-level logistics regression was used to estimate the crude and adjusted associations between exposure variables and non-attendance, adjusting for the a priori selected variables: sex, age and contact with the GP within the past year. Odds ratios (OR) and 95% confidence intervals (95% CI) are presented for all results.

## Results

### The Check-In intervention group and non-attendance

In total, 547 individuals were allocated to the Check-In intervention group. The median age was 54 years, 50% were employed and 79% had a Western background. In total, 42% were daily smokers, 13% exceeded the high-risk limit of alcohol consumption and 19% were obese (BMI ≥ 30). Moreover, 39% had fair or poor self-rated health, 54% had one or more metabolic risk conditions or non-communicable diseases (NCDs) and 90% had had contact with the GP within the past year (Table 1). In general, missing data were low; less than 5% of data from the questionnaires and equally distributed between attendees and non-attendees (data not shown).

**Table 1.** Characteristics for individuals allocated to the Check-In intervention group and divided by attendance status; *n*(%) if nothing else is stated.

	Check-In	Attendees	Non-attendees
	*n* = 547 (100)	*n* = 364 (67)	*n* = 183 (33)
**Demographic and socioeconomic characteristics**			
Age; median [IQR^2^;IQR^3^]	54 [49;58]	54 [49;58]	52 [48;57]
Male	281 (51)	183 (50)	98 (54)
Western origin	432 (79)	296 (81)	136 (74)
Living without partner	268 (49)	155 (43)	113 (62)
Affiliation to the labour market			
Employed	274 (50)	200 (55)	74 (40)
Unemployed or receiving social benefits	226 (41)	128 (35)	98 (54)
Retired or other	46 (8)	35 (9)	11 (6)
**Health behaviour**			
Daily smoker	228 (42)	135 (37)	93 (51)
Exceeding the high-risk limit (14/21)	71 (13)	50 (14)	21 (11)
BMI			
<25 kg/m^2^	222 (41)	148 (41)	74 (40)
25–29.9 kg/m^2^	196 (36)	130 (36)	66 (36)
≥30 kg/m^2^	104 (19)	73 (20)	31 (17)
Pulmonary symptoms (COPD PS score ≥3)	104 (19)	53 (15)	51 (28)
Perceived stress; median [IQR^2^;IQR^3^]	16 [12;21]	15 [11;20]	18 [14;24]
High stress (highest median split; scores 17–40)	243 (44)	143 (39)	100 (55)
Self-efficacy; median [IQR^2^;IQR^3^]	29 [24;33]	30 [25;33]	27 [22;32]
Low self-efficacy (lowest median split; scores 10–29)	272 (50)	168 (46)	104 (57)
Fair to poor self-rated health	213 (39)	118 (32)	95 (52)
**Morbidity and contact with GP**			
Number of metabolic risk conditions or non- communicable diseases			
0	251 (46)	172 (47)	79 (43)
1	140 (26)	99 (27)	41 (22)
2	82 (15)	47 (13)	35 (19)
≥3	74 (13)	46 (13)	28 (15)
Contact with the GP within the last year	493 (90)	341 (94)	152 (83)

A total of 183 individuals (33%) did not attend the preventive health check. Non-attendance was higher among men, individuals of non-Western origin and unemployed. More non-attendees compared to attendees reported daily smoking, higher pulmonary symptoms score, a high level of stress, low level of self-efficacy, had poor self-rated health, had two or more metabolic risk factor and NCDs and had had no contact with the GP within the past year (Table 1). Adjusted estimates showed that non-attendance was significantly associated with living without a partner, being of non-Western origin, daily smoking, fair or poor self-rated health, higher pulmonary symptoms score, high level of stress, low self-efficacy, metabolic risk factors and NCDs and no contact with the GP within the past year (Table 2).

**Table 2.** Crude and adjusted odds ratios (95% confidence intervals) for non-attendance to the prescheduled health check in the Check-In intervention group.

	Crude^a^	Adjusted^b^
	OR (95% CI)	OR (95% CI)
Age		
45–49	1	1
50–54	0.77 (0.48–1.25)	0.80 (0.49–1.30)
55–59	0.58 (0.35–0.95)	0.60 (0.36–0.99)
60–64	0.59 (0.34–1.02)	0.64 (0.36–1.11)
Sex		
Female (vs. male)	0.89 (0.62–1.29)	0.97 (0.67–1.42)
Country of origin		
Non-Western origin (vs. Western origin)	1.55 (1.00–2.41)	1.68 (1.07–2.65)
Cohabitation status		
Cohabitant (vs. single)	0.42 (0.28–0.61)	0.43 (0.29–0.63)
Affiliation to the labour market		
Employed	1	1
Unemployed or receiving social benefits	2.14 (1.45–3.16)	2.32 (1.54–3.49)
Retired or other	0.83 (0.40–1.75)	0.88 (0.37–2.06)
Daily smoker		
Yes (vs. no)	1.81 (1.25–2.64)	1.70 (1.15–2.50)
Exceeding the high-risk limit (14/21)		
Yes (vs. no)	0.80 (0.46–1.39)	0.73 (040–1.31)
BMI		
<25 kg/m^2^	1	1
25–29.9 kg/m^2^	1.02 (0.68–1.56)	1.09 (0.70–1.69)
≥30 kg/m^2^	0.82 (0.49–1.37)	0.97 (0.57–1.65)
Pulmonary symptoms score		
≥3 (vs. 0–2)	2.18 (1.40–3.40)	2.37 (1.50–3.75)
Perceived stress (PSS)		
Highest split (vs. lowest split)	2.15 (1.46–3.18)	2.24 (1.49–3.37)
Self-efficacy		
Highest split (vs. lowest split)	0.56 (0.38–0.82)	0.50 (0.33–0.76)
Self-rated health		
Excellent/very good/good (vs. fair/poor)	0.44 (0.34–0.64)	0.38 (0.26–0.57)
Metabolic risk conditions or non-communicable diseases		
Yes (vs. no)	1.13 (0.78–1.63)	1.53 (1.01–2.30)
Contact with the GP within the last year		
Yes (vs. no)	0.32 (0.18–0.57)	0.32 (0.18–0.59)

a ICC = (0.13)/(0.13 + 3.359) × 100% ≈ 3.7%.

b Adjusted for sex, age and contact with general practitioner within the last year.

### Non-responders

From the registers we found that 3873 had lower secondary school and 1573 of these responded, meaning that we reached 41% of the target group. Non-respondents were more likely to be male, unemployed and live without a partner. Further, more of the non-respondents had no metabolic risk factors or NCDs and no contact to the GP within the past year (Supplementary 2 and 3).

## Discussion

### Principal findings

Overall, 33% of the individuals invited to the preventive health checks did not attend. Non-attendees were more likely to live without a partner, be of non-Western origin, be daily smokers, have higher pulmonary symptoms score, have poor or fair self-rated health, have high level of stress, have low self-efficacy and no contact with the GP within the past year.

### Strengths and weaknesses

The major strengths of this study include the combination of data from the questionnaires and the access to data from valid, high-quality national registers on SEP and health, which ensured information on non-responders. Furthermore, availability of register-based data meant that eventually missing data was not related to attendance status [14]. The study design with the baseline questionnaire before the health check ensured a large amount of data – even on non-attendees. Missing data occurred in the questionnaires, which could lead to information bias. Nevertheless, missing data were low and the equally distributed between attendees and non-attendees, indicating that missing was not associated with attendance status. A limitation of the study is the fact that the questionnaire and the invitation to the health check were only available in Danish, which could be the main reason for the lower response and attendance rates for individuals with non-Western origin. Another limitation is the fact that only individuals who answered the questionnaire from the GP could be invited to the health check; in this way a self-selection occurred which could affect and lower the non-attendance rate compared to other studies. Further, this means that individuals, who cannot manage to answer a questionnaire from the GPs, i.e. due to few physical or psychological resources, low literacy or no sufficiency in the Danish language, are not reached and recruited in the Check-In RCT. Paradoxically, this group can be expected to be those most likely to benefit from preventive health checks. From the registers, we found, however, that more non-responders compared to responders had no metabolic risk factors or non-communicable diseases (NCDs), indicating either that the non-responders were a healthier group or that the non-responders were more likely to have undiagnosed metabolic risk factor or NCDs.

### Findings in relation to other studies

A successful recruitment strategy for preventive health checks needs to fulfil at least two conditions. First, it should be able to identify those in need of health checks and second, it should result in high attendance. Different approaches have been used to identify those in need, including the use of medical health record information [24], combined with an area-based deprivation score [25] or limiting the invitation to socially deprived areas [26]. In the present study we used low level of education as measure for low SEP. Educational level captures the influence of resources on health and the knowledge and skills attained through education may affect an individual’s cognitive functioning, make them more receptive to health education messages, or more able to communicate with and access appropriate health services [27]. The characteristics of the individuals in the Check-In intervention group showed that more than 42% were daily smokers as compared to 17% in the general Danish population [28] and 30% in previous studies of health checks [29]. As regards alcohol consumption, 13% exceeded the high-risk limit compared to 7% in the general Danish population [28]. This indicates that the individuals recruited for the Check-In RCT had more adverse health behaviour profiles than the general population and may for that reason, as a group, be more in need of health checks. In the Check-In RCT, only individuals with low levels of education were invited to the health check and an extra effort was made to reach these individuals. However, even in this relatively homogeneous target group, in terms of SEP, we found differences between attendees and non-attendees. In line with previous studies we found that non-attendees were more likely to live without a partner [2] and be of non-Western origin [1,4]. Further, daily smokers and individuals with low self-efficacy were less likely to attend the health check, which correspond to previous studies [4,30]. Taken together, these findings indicate that the inverse care law also applies to the uptake of preventive health checks when individuals with low SEPs are targeted with proactive recruitment by GPs.

In the present study the non-attendance rate was 33% of the invited individuals, which is low compared to 70–75% in other studies targeting high-risk groups [26,31]. However, the possible self-selection described under limitations is important to keep in mind when comparing the non-attendance rates. Nevertheless, the lower non-attendance seen in our study might be a result of the proactive recruitment by the GPs and the use of the general practice as the setting, rather than municipalities or a unit specifically designed by researchers, which are used in many other studies of health checks [2,26]. A Dutch study in which GPs were also actively used in the recruitment of individuals with low SEPs found a non-response rate more similar to ours, at 38% [31]. These findings correlate well with a recent qualitative study of individuals in the Check-In RCT, which found that individuals were motivated to attend health checks when invited by their GP [32].

### Meaning of the study

Individuals enrolled in the Check-In RCT had more adverse health behaviour profiles than the general population and non-attendees had worse overall health compared to attendees. This points to the fact that besides targeting high-risk groups, improved efforts to increase the uptake from these more in-need groups are still necessary to avoid the possibility that health checks exacerbate rather than narrow social inequalities in access to prevention programmes and eventually to inequalities in health. The question remains what the best strategy is for reaching individuals in need of a health check. Should this be a strategy based on SEP, medical record/risk assessment, deprived areas or a fourth strategy? In the present study, it is noteworthy that even though one out of three individuals did not attend the prescheduled preventive health check, 37% of those who attended were smokers, 14% exceeded the high-risk limit regarding alcohol consumption and 20% were obese. This indicates that health interventions were amenable. On the other hand, using the GPs in the recruitment made it difficult to reach those who had had no contact with the GP within the past year, suggesting that a single recruitment strategy does not fit all individuals and that complementary recruitment strategies may reach the non-attendees in this study.

In conclusion, the recruitment strategy was successful regarding the low non-attendance rate and the adverse health behaviour profile of those attending. The findings suggest that it is feasible to use the GPs proactively in the reach and recruitment of individuals with low SEP to preventive health checks. It is, however, important to acknowledge that even in a selected group of individuals with low level of education there were differences between the attendees and non-attendees. Non-attendees being more disadvantaged both regarding SEP and health. It is important to acknowledge and address disparities in socioeconomic and health when it comes to reach and recruitment to preventive initiatives such as preventive health checks.

## Supplementary Material

Supplemental Material

## Registration number

The Check-In randomised controlled trial is registered at ClinicalTrials.gov (Early detection of and intervention towards chronic diseases; ID NCT01979107; October 25, 2013).
