# Supplementary material for: Using general practitioners to recruit individuals with low socioeconomic position to preventive health checks is feasible: a cross sectional study
Source: Scand J Prim Health Care. 2019 Jul 9;37(3):294–301. doi: 10.1080/02813432.2019.1639901 (PMC6713093; doi:10.1080/02813432.2019.1639901)
Supplement: Supplemental Material [file IPRI_A_1639901_SM3413.docx]

**Supplementary material**

**Supplementary 1.** Items in the baseline questionnaire, response categories and new classifications and scores when applied.

| **Item** | **Response** |  |
| --- | --- | --- |
| **Identifying the study population** | | |
| What school education do you have? | 7 or fewer years of schooling  8-9 years of schooling  10-11 years of schooling  Upper Secondary School Leaving Examination or similar | Enrolled  Enrolled  Enrolled  Not enrolled |
| Have you completed an occupational education? | No  Enrolled in education  Semi-skilled worker  Vocational education  Short further education (fewer than 3 years)  Short further education (3-4 years)  Higher education (more than 4 years)  Other education | Enrolled  Not enrolled  Enrolled  Not enrolled  Not enrolled  Not enrolled  Not enrolled  Not enrolled |
| **Pulmonary symptoms^#^** |  | **Score** |
| During the past 4 weeks, how much of the time did you feel short of breath? | None of the time | 0 |
|  | A little of the time | 0 |
|  | Some of the time | 1 |
|  | Most of the time | 2 |
|  | All of the time | 2 |
| Do you ever cough up any “stuff,” such as mucus or phlegm? | No, never | 0 |
|  | Occasionally / only when I have a cold | 0 |
|  | Few days a month | 1 |
|  | Most days a week | 1 |
|  | Yes, every day | 2 |
| Please select the answer that best describes you in the past 12 months: I do less than I used to because of my breathing problems. | Strongly disagree | 0 |
|  | Disagree | 0 |
|  | Unsure | 0 |
|  | Agree | 1 |
|  | Strongly agree | 2 |

^#^ Items from the COPD Population Screener developed by Martinez and colleagues [21]

**Supplementary 2**. Register-based educational level of the total invited population and divided by response status.

|  | Total population  n=17,063 (100) | Respondent  n=8,377 (49) | Non-respondent n=8,686 (51) |
| --- | --- | --- | --- |
| Highest completed education  Lower secondary school  Upper secondary school  Vocational education  Short education  Medium education  Long education  Missing/non-classifiable | 3,873 (23)  1,321 (8)  4,803 (28)  792 (5)  3,025 (18)  2,175 (13)  1,074 (6) | 1,573 (19)  629 (8)  2,486 (30)  400 (5)  1,797 (21)  1,233 (15)  259 (3) | 2,300 (26)  692 (8)  2,317 (27)  392 (5)  1,228 (14)  942 (11)  815 (9) |

**Supplementary 3**. Register-based characteristics for individuals with lower secondary school education (obtained from register) of the total invited population and divided by response status; n(%) if nothing else is stated.

|  | Lower secondary school  N=3,873 (100) | Respondent  N=1,573 (41) | Non-respondent  2,300 (59) |
| --- | --- | --- | --- |
| Age; median [IQR^2^;IQR^3^] | 52 [48;57] | 53 [49;58] | 52 [47;57] |
| Male | 2,117 (55) | 795 (51) | 1,322 (57) |
| Western origin | 3,078 (79) | 1,308 (83) | 1,770 (77) |
| Living without partner | 2,000 (57) | 821 (52) | 1,379 (60) |
| Affiliation to the labour market  Employed  Unemployed or receiving social benefits  Retired and other | 1,881 (49)  1,716 (44)  275 (7) | 865 (55)  594 (38)  113 (7) | 1,016 (44)  1,122 (49)  162 (7) |
| Number of metabolic risk conditions and non-communicable diseases  0  1  2  ≥3 | 2,248 (53)  1,126 (27)  540 (13)  292 (7) | 821 (43)  523 (27)  320 (17)  242 (13) | 1,427 (62)  603 (26)  220 (10)  50 (2) |
| Number of contacts with GP within the past year  0  1  2-4  ≥ 5 | 604 (16)  321 (8)  775 (20)  2,173 (56) | 189 (12)  116 (7)  320 (20)  948 (60) | 415 (18)  205 (9)  455 (20)  1225 (53) |
